# Supplementary material for: Amyloid Formation in Nanoliter Droplets
Source: Int J Mol Sci. 2022 May 13;23(10):5480. doi: 10.3390/ijms23105480 (PMC9143811; doi:10.3390/ijms23105480)
Supplement: Supplementary file 1 [file ijms-23-05480-s001.zip › ijms-1681373-supplementary.pdf]

Supplementary figures:

Amyloid Formation in Nanoliter Droplets

Da Yeon Cheong[1,2,†], Wonseok Lee[3,†], Insu Park[4], Jinsung Park[5,*], and Gyudo Lee[1,2,*]

[1]Department of Biotechnology and Bioinformatics, Korea University, Sejong 30019, Korea
[2]Interdisciplinary Graduate Program for Artificial Intelligence Smart Convergence Technology, Korea University, Sejong 30019, Korea
[3]Department of Electrical Engineering, Korea National University of Transportation, Chungju 27469, Korea
[4]Nick Holonyak Jr. Micro and Nanotechnology Laboratory, University of Illinois at Urbana-Champaign, Urbana, IL 61801, USA
[5]Department of Biomechatronic Engineering, College of Biotechnology and Bioengineering, Sungkyunkwan University, Suwon 16419, Korea
*Correspondence: nanojspark@skku.edu (J.P.); lkd0807@korea.ac.kr (G.L.)
[†]These authors contributed equally to this work.

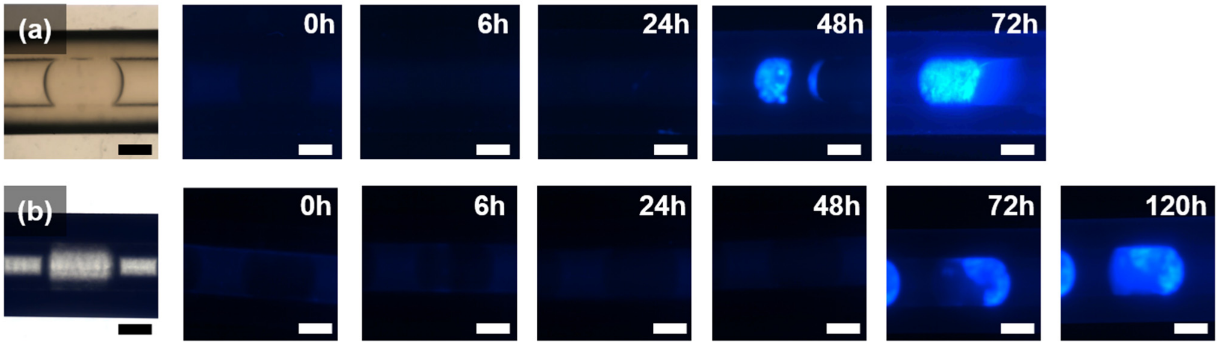

Figure S1. Amyloid fibrillation in nanoliter droplets. Bright-field images of nanoliter droplets depend on protein concentration ((a) 3 wt% HEWL (b) 1 wt% HEWL), and corresponding fluorescence images of nanoliter droplets during incubation. The droplet volume for (a) and (b) is 239.7 nL and 237.8 nL, respectively. (Scale bar: 500 μm).

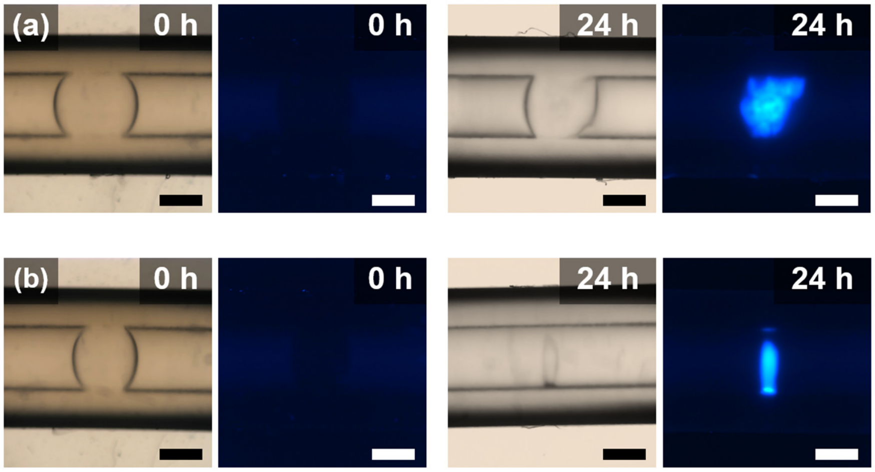

Figure S2. Droplet shrinking phenomenon. (a) 188.7 nL droplet (b) 147.9 nL droplet.
